# Lifestyle Outcomes Six and Twelve Months After Hypertensive Disorders of Pregnancy: A Blood Pressure Postpartum Sub-Study

**DOI:** 10.3390/nu18040610

**Published:** 2026-02-12

**Authors:** Jenny Zhang, Lynne Roberts, Kaylee Slater, Justine Salisbury, Megan Gow, Amanda Henry

**Affiliations:** 1 Discipline of Paediatrics and Child Health, School of Clinical Medicine, UNSW Medicine and Health, Sydney, NSW 2031, Australia; jenny.zhang1@student.unsw.edu.au; 2 Department of Women’s and Children’s Health, St George Hospital, Sydney, NSW 2217, Australia; lynne.roberts2@health.nsw.gov.au; 3 St. George and Sutherland Clinical Campus, School of Clinical Medicine, UNSW Medicine and Health, Sydney, NSW 2217, Australia; 4 School of Health Sciences, Faculty of Medicine and Health, University of Sydney, Sydney, NSW 2006, Australia; kaylee.slater@sydney.edu.au; 5 Ministry of Health NSW, St. Leonards, NSW 2065, Australia; justine.salisbury@health.nsw.gov.au; 6 The George Institute for Global Health, University of New South Wales, Sydney, NSW 2031, Australia; 7 School of Population Health, UNSW Medicine and Health, Sydney, NSW 2031, Australia; 8 Discipline of Women’s Health, School of Clinical Medicine, UNSW Medicine and Health, Sydney, NSW 2031, Australia

**Keywords:** hypertensive disorders of pregnancy, postpartum, diet, physical activity, lifestyle

## Abstract

**Background/Objectives**: Hypertensive disorders of pregnancy (HDP) increase the risk of cardiovascular disease (CVD), but few studies have explored the efficacy of lifestyle interventions to improve CVD risk post-HDP. This study compared the 6 month (6M) and 12 month (12M) dietary and physical activity outcomes of women post-HDP participating in one of three lifestyle interventions. **Methods**: This sub-study of the Blood Pressure Postpartum (BP^2^) randomised controlled trial included participants from six hospitals across Sydney, Australia, randomly assigned to one of three groups: Group 1 (usual care) received general postpartum health information; Group 2 (brief education) received usual care plus an individualised cardiovascular risk assessment and lifestyle counselling; Group 3 (extended lifestyle) received all Group 2 components plus enrolment in a six-month telephone coaching programme. Baseline and post-intervention data were collected at 6M and 12M, respectively. Diet and physical activity were assessed using the NSW Population Health Survey, alongside cardiometabolic measures. **Results**: Overall, 405 women provided complete 6M and 12M data (Group 1 *n* = 129, Group 2 *n* = 137, Group 3 *n* = 139). From 6M to 12M, Group 3 increased their vegetable serves/day (3.0 vs. 2.0, *p* = 0.001). No significant changes in fruit intake and physical activity levels were observed among groups. Groups 2 and 3 reported that nutritional information had a greater influence on their food choices at 12M (*p* = 0.010 and *p* < 0.001, respectively). At 12M, higher vegetable and fruit intake correlated with lower body mass index (BMI) (*p* = 0.006, *p* = 0.003) and waist circumference (*p* = 0.035, *p* = 0.014), and increased vigorous and strength exercise correlated with lower BMI (*p* = 0.005, *p* = 0.003) and waist circumference (*p* < 0.001, *p* < 0.001). **Conclusions**: Intensive lifestyle interventions improved vegetable intake and nutrition awareness in post-HDP women at 12M, holding promise for long-term cardiometabolic health benefits.

## 1. Introduction

Hypertensive disorders of pregnancy (HDP), including preeclampsia (PE), gestational hypertension (GH), and chronic hypertension (CH) affect approximately 10% of pregnancies worldwide [1]. Following HDP, women have a 3–4 times increased risk of chronic hypertension and a 2–3 times increased risk of cardiovascular disease (CVD), including ischaemic heart disease, stroke, and cardiovascular death [2,3,4,5,6,7]. CVD is the leading cause of death in Australian women, accounting for 30% in 2016 [8]. Adverse cardiovascular outcomes following HDP are a major public health concern, and evidence for effective interventions to alter this trajectory is urgently needed.

The exact pathophysiology of CVD following HDP remains unclear; it is theorised that the physiological stressors of pregnancy may reveal the woman’s latent CVD risk, or HDP may negatively alter maternal vasculature, or both [9]. Emerging evidence suggests that angiogenic imbalance during pregnancy may contribute to subtle long-term vascular and metabolic alterations [10].

Lifestyle risk factors such as obesity, poor diet, physical inactivity, smoking, and excess alcohol intake are well-established contributors to CVD, and early intervention to address these can significantly reduce long-term CVD risk [8,11]. Women with a history of HDP are more likely to have modifiable risk factors—such as high body mass index (BMI), high-risk alcohol use, and depressive symptoms—placing them at greater risk of future CVD [12,13]. Therefore, implementing lifestyle interventions in the postpartum period offers a critical opportunity to improve these behaviours and mitigate long-term risk. However, despite guideline recommendations [14,15,16] that mirror general CVD prevention recommendations (e.g., weight loss, exercise, diet), there remains limited evidence specific to HDP populations demonstrating that such interventions are effective in modifying lifestyle factors or reducing CVD risk.

The aim of this study was to evaluate the efficacy of three postpartum lifestyle interventions of varying intensity in women with a history of HDP. The primary objective was to determine whether lifestyle interventions result in improvements in dietary quality and physical activity levels at twelve months postpartum. A secondary objective was to assess whether improvements in diet and physical activity are associated with improvements in established cardiovascular risk markers, specifically body mass index (BMI) and blood pressure (BP).

## 2. Materials and Methods

This project was a sub-study of Blood Pressure Postpartum (BP^2^), a 3-arm randomised controlled trial (RCT) aiming to compare outcomes of follow-up and lifestyle management strategies in the first twelve months following HDP [17]. The full methodology and primary results have been previously published [17,18]. In brief, BP^2^ was conducted across six metropolitan Sydney hospitals servicing a socio-demographically diverse population with approximately 20,000 births per year. Study participants were randomly assigned 1:1:1 to one of three trial groups:

1. Optimised usual care (control): Women received general information about health following HDP and were instructed to see their General Practitioner (GP) for BP and health check-up six months postpartum (6M).
2. Brief education intervention: In addition to receiving the Group 1 information pamphlets, women attended a specialised postpartum clinic to receive an individualised assessment of personal cardiovascular risk profile from a study physician, and diet and exercise education from a study dietician.
3. Extended lifestyle intervention: In addition to Group 2, women were enrolled into a 6-month lifestyle behaviour change service (Get Healthy Service) with up to 10 coaching phone calls over a 6-month period. This service was delivered by trained dietitians and exercise physiologists.

### 2.1. Eligibility and Recruitment

Eligible women were aged ≥ 18 years and had a pregnancy complicated by either CH, GH, PE, or PE superimposed on CH (PE+CH). Exclusion criteria included stillbirth or neonatal death, a severe mental health condition or developmental disability preventing informed consent. Eligible women were contacted 5 months postpartum to confirm consent, and baseline data were collected via questionnaires both online and by telephone, using interpreter services as required. Randomisation was stratified by hospital, parity 1 or > 1, and BMI < 30 or ≥ 30. Due to the nature of the intervention, participants were unable to be blinded to allocation.

### 2.2. Sub-Study Data Collection

Baseline data were collected at 6M (accepted range 5–8 months postpartum) and post-intervention at 12M (accepted range 11–15 months postpartum) by trained staff. Lifestyle behaviours were assessed using a modified New South Wales (NSW) Population Health Survey [19]. These questions were completed via telephone, which is the format for which they were developed. Women self-reported on diet (fruit and vegetable serves, meat products, hot fried potato products, salty snack products, milk, cordial/juice, fast food consumption, cups of water), physical activity (walking, vigorous chores, gardening/heavy yard work, moderate and vigorous exercise, strength exercise), nutrition awareness and alcohol consumption. Pregnancy details such as HDP subtype, infant birthweight and postpartum complications were recorded from the hospitals’ maternity database. 

Other data collected at 6M and 12M included weight, height, waist circumference and BP. BP was measured using an average of three measurements from a validated automated device (Omron HEM-907, Omron Corporation, Kyoto, Japan). Clinical staff taking physical measurements were blinded to group allocation.

### 2.3. Sub-Study Outcomes

This sub-study focused on maternal diet and physical activity outcomes at 6M and 12M. Primary outcomes were vegetable serves/day, fruit serves/day and time spent participating in physical activity/week. New variables were created to identify participants meeting the recommended 5 vegetable serves/day, 2 fruit serves/day, and 150 min of moderate-vigorous exercise/week. Physical activity was calculated as the sum of time spent walking, moderate exercise, strength training, gardening or heavy yard work, and vigorous activities. To reflect their higher intensity, minutes spent on vigorous activities were weighted by a factor of two [20].

### 2.4. Statistical Analysis

Analysis was conducted using IBM SPSS Statistics Version 27. Descriptive statistics were used to describe women’s demographic characteristics and lifestyle outcomes at 6M and 12M, with continuous parametric data expressed as mean ± standard deviation, continuous non-parametric data as median [interquartile range] and categorical data as number (percentage). *p*-values were calculated using Pearson Chi-Square for categorical values and ANOVA test for continuous variables. Outcomes were compared across time using McNemar’s test for paired categorical data and the Wilcoxon signed-rank test for paired non-parametric continuous data. Spearman’s correlation was used to assess the association between changes in diet and exercise outcomes and changes in cardiometabolic measures. Subgroup analysis was conducted to assess whether these correlations differed by baseline BP, stratified into normotensive (systolic blood pressure [SBP] < 140 mmHg and diastolic blood pressure [DBP] < 90 mmHg) and hypertensive (SBP ≥ 140 mmHg or DBP ≥ 90 mmHg) groups. Subgroup analysis was also performed by baseline BMI category, stratified into BMI < 25 kg/m^2^, BMI 25–29.9 kg/m^2^, and BMI ≥ 30 kg/m^2^. Statistical significance was set at *p* < 0.05.

### 2.5. Ethics Approval

The study was approved by the South-Eastern Sydney Local Health District (Ref: 18/193) and prospectively registered in the Australian and New Zealand Clinical Trials Registry (ACTRN12618002004246).

## 3. Results

**Figure 1:**
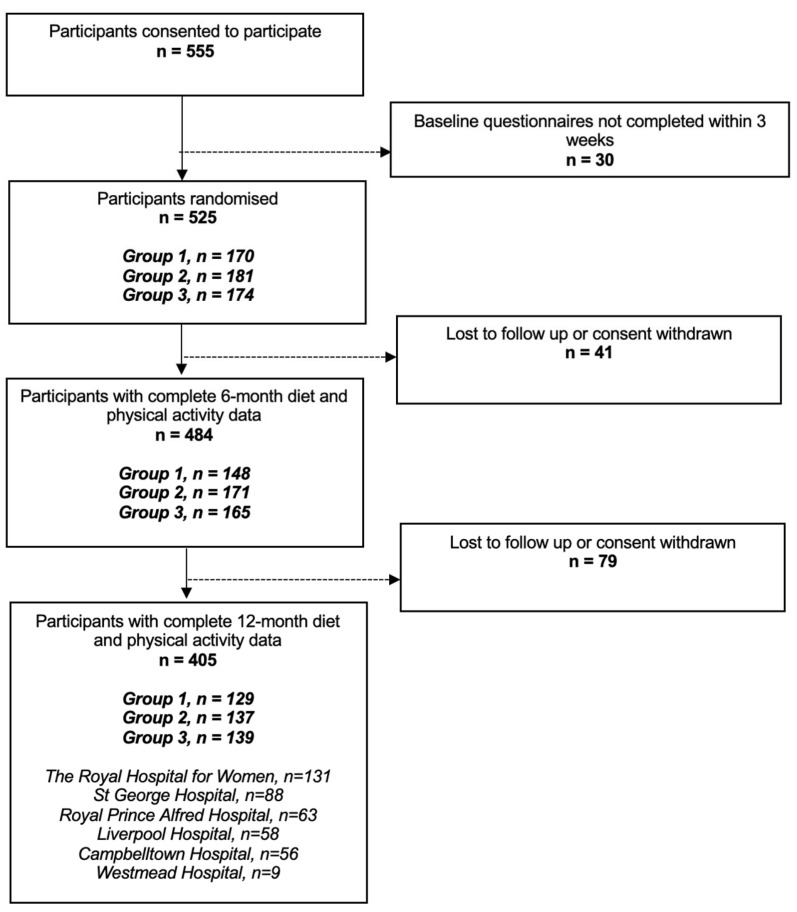
Participant flow diagram for lifestyle sub-study of BP^2^.

Of the 555 women who consented for BP^2^, 405 had complete diet and physical activity data at 6M and 12M postpartum: 129 were assigned to Group 1, 137 to Group 2, and 139 to Group 3. Figure 1 shows the recruitment and final number of participants in this sub-study.

### 3.1. Study Population Characteristics

Of the 405 women with complete 6M and 12M diet and physical activity data, 65 (16%) had CH, 93 (23%) had GH, 225 (56%) had PE and 22 (5%) had PE+CH. Table 1 describes participant demographics and details of the index pregnancy between intervention groups.

The average maternal age at 6M was 34.9 ± 5.1 years, and participants were predominantly Caucasian (63%) and born in Australia (60%). Most participants were highly educated, with 70% holding a university degree. In the majority of cases (93%), the woman’s current partner was the father of the index baby. Nearly all pregnancies were singleton (97%), and 65% of women were breastfeeding at 6 months postpartum. The median maternal postnatal hospital length of stay was 6 days [IQR: 5–8], and 16% of women required a postnatal admission to acute care. Overall, there were no differences in participant demographics, details of index pregnancy and HDP subtype between the three groups.

### 3.2. Lifestyle Outcomes

The lifestyle outcomes at 6M and 12M, as collected from the NSW Population Health Survey, are shown in Table 2. Outcomes by HDP subtype can be found in Supplementary Table S1.

From 6M to 12M, median vegetable intake increased modestly from 2.0 to 2.5 serves/day (*p* = 0.03), driven primarily by Group 3 participants, who reported a significant improvement from 2.0 to 3.0 serves/day (*p* = 0.001). No significant change was observed in Groups 1 or 2. Despite these improvements, only 9% of participants met the recommended five serves/day at 12 months (*n* = 35).

Fruit intake remained unchanged across the cohort, with a median of 1.0 serve/day (IQR 1.0–2.0) at both time points, and no significant changes were detected within groups. At 12M, less than half of the women were meeting the recommended two fruit serves/day (47%, *n* = 189).

Intake of processed meat, hot fried potato products and salty snacks decreased significantly overall (*p* < 0.001), with Groups 2 and 3 showing the largest reductions. These groups also reported increased influence of nutrition information on food purchases at 12M (Group 2: *p* = 0.010; Group 3: *p* < 0.001). Overall, at 12M, 54% of participants (*n* = 220) indicated that nutrition information influenced their choices “a great deal” (*p* < 0.001).

Physical activity levels remained unchanged, with an overall median of 280 min/week of moderate-vigorous activity at both 6M and 12M (IQRs 140–465 and 150–450, respectively), and no significant changes were observed in any group. At 12M, 76% of participants (*n* = 305) were meeting the recommended 150 min/week of moderate-vigorous activity. Most activity came from walking, with minimal time spent on structured exercise or strength training.

### 3.3. Cardiometabolic Risk Factors

Correlation analysis was performed to assess whether changes in diet and physical activity were associated with changes in cardiometabolic risk factors from 6 to 12 months postpartum (Supplementary Table S2). No significant correlations were observed between any change in lifestyle outcome and change in SBP, DBP, or BMI.

Subgroup analyses examined whether correlations between lifestyle changes and changes in cardiometabolic outcomes varied by baseline risk. When stratified by baseline (6M) BP (Table 3), significant associations were observed only in women with hypertension (SBP ≥ 140 mmHg or DBP ≥ 90 mmHg at baseline). In this group, increased vegetable intake was associated with reductions in both SBP (r = −0.35, *p* < 0.01) and DBP (r = −0.26, *p* < 0.05). No significant correlations were observed in the normotensive group. Analysis stratified by baseline BMI (Table 4) revealed that among women with BMI < 25 kg/m^2^, increased total moderate-vigorous activity was weakly associated with lower DBP (r = −0.18, *p* < 0.05).

## 4. Discussion

### 4.1. Summary of Main Findings

Overall, only small improvements in diet and physical activity outcomes were observed in women post-HDP from 6M to 12M postpartum. The more intensive lifestyle intervention that women in Group 3 participated in was associated with a significant increase in daily vegetable servings; however, no significant change in fruit intake or physical activity was found in any group.

### 4.2. Diet

At 12M, only 9% of women were meeting the recommended 5 servings of vegetables per day, and 47% were meeting the recommended 2 servings of fruit per day. These results are consistent with our previously published 6M results [21]. Our findings are also comparable to previous studies showing inadequate maternal dietary intake during the early postpartum period [22,23,24,25,26], including a Melbourne-based study of first-time mothers (*n* = 448), which found that 8.6% of women were meeting vegetable recommendations and 55.4% were meeting fruit recommendations [22]. Given the well-established relationship between poor diet and CVD, it is vital that future interventions target increasing vegetable and fruit intake in the post-HDP population.

Participants in the most intensive intervention group, Group 3, achieved a median increase of one daily vegetable serve (*p* = 0.001) from 6M to 12M. This is significant as studies have shown that even modest increases in vegetable intake have been associated with reduced risk of ischaemic heart disease [27,28] and stroke [29]. These benefits are particularly significant for individuals with very poor vegetable intake; a recent meta-analysis found that a vegetable intake of 100g/day compared to 0g was associated with a 19.8% lower risk of ischaemic stroke and a 19.3% lower risk of ischaemic heart disease [30]. Fruit intake increased for Group 3 (1 to 1.4 daily serves) but not Groups 1 or 2. While this finding was not statistically significant, it suggests that extended lifestyle interventions are more likely to produce change compared to information packages or a one-off counselling session. Promisingly, at 12M, both Groups 2 and 3 reported that nutritional information had a greater influence on their food choices compared to at 6M (*p* = 0.010 and *p* < 0.001, respectively), suggesting an increased knowledge gain in women receiving these more intensive educational interventions.

The weekly intake of processed meat, hot fried potato products and salty snacks decreased significantly from 6M to 12M (*p* < 0.001); however, these overall small changes may not be considered clinically meaningful and were not different between groups.

### 4.3. Physical Activity

No significant changes in physical activity were observed in any intervention group within the BP^2^ study. This aligns with the Heart Health 4 Moms RCT, which also found no difference in physical activity between intervention and control arms among women with recent PE [31]. In contrast, a 2022 gamified text-based intervention in post-HDP women significantly increased daily step count in the intervention group compared to the control group (647 steps, *p* = 0.009) [32], likely due to the motivational impact of step tracking. A more recent large-scale RCT of 619 women with prior HDP tested two app-based physical activity interventions incorporating behaviour change techniques but found no effect on physical activity levels [33]. Notably, only 15% of participants were enrolled within 12 months postpartum, and baseline physical activity was already high, potentially limiting intervention impact. These findings suggest that interventions relying on education or behavioural theory alone may be insufficient, and more immediate, feedback-driven strategies such as gamification may better promote physical activity in this population.

At 12M, 76% of women were meeting the recommended 150 min/week of moderate-vigorous activity, with a median of 280 min/week. This is lower than the reported activity levels of participants in van der Pligt et al.’s Melbourne-based study on general postpartum women, which recorded a mean total physical activity time of 351 min/week [22]. This difference may indicate the post-HDP population is less active than the general postpartum population, which is concerning, as physical activity is a protective factor for CVD. Like in our study, van der Plight et al.’s total physical activity time was calculated from the sum of walking, moderate physical activity, and twice the time spent on vigorous activity; however, household chores, gardening, and yard work were excluded. Van der Pligt et al.’s data was collected at three months postpartum compared to our results at 12M postpartum; however, given that studies have shown that physical activity levels tend to remain unchanged in the postpartum period [34,35], this does not explain their significantly higher value. Our results are comparable with a cross-sectional study conducted in Tokyo, Japan (*n* = 110), where at 2 months postpartum, the average time spent on physical activity was 240 min/week [36] as recorded by an accelerometer. Similarly, a cohort study in the United States (*n* = 204) using both accelerometers and self-reported measures found at 12 months postpartum, the average time spent on physical activity was 282 min/week [37]. Unlike our study, these studies did not specifically focus on women with a history of HDP. Together, these findings suggest that post-HDP women may have lower activity levels than the general postpartum population, underscoring the need for targeted interventions to support sustained physical activity in this high-risk group.

Walking made up most physical activity at both 6M and 12M. This is in line with previous studies, which found walking to be the most common type of exercise in postpartum women [38,39]. This study was also conducted during the COVID-19 pandemic, which impacted available exercise types and led to a reduction in Australians’ participation in team sports and gyms, while home exercise and recreational walking remained unchanged [20]. A previously published finding from this trial was that participants were more likely to do any walking, vigorous activity and strength training prior to COVID-19, compared to during or after COVID-19-related lockdowns [40]. Walking in combination with improvements in dietary intake has been shown to significantly decrease BMI in women by 6 months postpartum [41]. Walking is a cost-effective and accessible form of exercise, and therefore can be used to increase physical activity levels in the post-HDP population.

### 4.4. Correlation Between Changes in Lifestyle Factors and Cardiometabolic Risk Factors

In contrast to previous cross-sectional findings at 6 months postpartum [21], the present study did not identify significant associations between changes in lifestyle behaviours and changes in cardiometabolic outcomes in the overall sample. The earlier analysis reported strong correlations between healthier diet and physical activity patterns and more favourable cardiometabolic profiles, including reduced BMI, waist circumference, fat mass, and BP [21]. However, in this longitudinal analysis from 6 to 12 months postpartum, lifestyle changes over time were not significantly associated with changes in BMI, SBP or DBP when analysed across the whole cohort. Although the intervention produced modest improvements in vegetable intake and nutrition awareness at 12 months, it is uncertain whether such relatively small behavioural changes are sufficient to produce measurable cardiometabolic benefits within the short 6-month follow-up period. This may partly explain why no significant associations were detected at the cohort level, with correlations emerging only among specific higher-risk subgroups. Notably, subgroup analyses suggested that such associations may be more evident among women at higher baseline risk. Among women with hypertension at baseline, increased vegetable intake was associated with reductions in both SBP and DBP, while in women with a healthy baseline BMI, greater total physical activity time was weakly associated with lower DBP. A broader consideration of potential biological mechanisms—such as differential vascular reactivity or persistent endothelial dysfunction following HDP—may help contextualise why certain subgroups demonstrated stronger associations. The differences between the cross-sectional and longitudinal analyses may also reflect the challenges in sustaining behaviour change over time, the modest magnitude of changes achieved, or the possibility that more intensive interventions are needed to elicit measurable cardiometabolic improvements postpartum.

### 4.5. Strengths, Limitations, and Future Directions

This study is one of few RCTs investigating the effect of various postpartum lifestyle interventions in improving diet and physical activity outcomes in women following HDP. Education was delivered in the form of handouts and consultations with healthcare professionals; this has been previously identified by Australian women as an appropriate method of delivering information regarding long-term health following HDP [42]. To our knowledge, this study is the first to describe how changes in lifestyle behaviours correlate with changes in cardiometabolic outcomes in the post-HDP population. Our large sample size with socioeconomic and culturally diverse participants increases the generalisability of results to the broader post-HDP population in Australia. However, a key limitation is the reliance on self-reported measures of diet and physical activity, which are subject to recall bias. Additionally, underreporting of dietary intake is particularly common in the overweight population [43]. Diet and physical activity outcomes were assessed using validated questions from the NSW Population Health Survey, but objective measures (e.g., accelerometry or dietary biomarkers) may have provided more accurate estimates. Additionally, Indigenous Australian women were underrepresented in this cohort, despite their significantly higher burden of chronic disease and unique barriers to lifestyle modification [44]. Future research should prioritise culturally safe, community-led approaches to address the needs of this population.

Despite not finding significant improvements in fruit intake or physical activity, we noted that the intervention of greatest intensity (a 6-month lifestyle phone coaching intervention) showed promise in improving a variety of diet and physical activity-related outcomes. While diet and physical activity improvements from the interventions delivered in this study are small, such small changes may lead to meaningful improvements in future CVD risk, suggesting that, at a minimum, post-HDP women should receive some form of specific education to encourage healthy lifestyle change in the early postpartum period. Women should also be encouraged to attend appointments with their primary health provider to help maintain positive lifestyle change. Overall, RCTs on lifestyle-based interventions for the post-HDP population are limited and the sustainability of behavioural lifestyle change is unclear. Further large studies with targeted interventions and extended follow-ups are needed to assess whether long-term cardiovascular risk is reduced.

## 5. Conclusions

HDP is common, and the long-term cardiovascular risks are serious. This study has shown that a 6-month lifestyle behaviour change service improves vegetable intake but not fruit intake or physical activity levels in the post-HDP population. The results of this study carry public health significance and can inform the development of future interventions to improve lifestyle behaviours and reduce the risk of CVD in the post-HDP population.

## Figures and Tables

**Table 1:** Participant demographics and details of index pregnancy by intervention group.

	Total (*n* = 405)	Group 1: Optimised Usual Care (*n* = 129)	Group 2: Brief Education Intervention (*n* = 137)	Group 3: Extended Lifestyle Intervention (*n* = 139)	*p*-Value *
**HDP subtype**					0.58
Chronic hypertension	65 (16)	17 (13)	22 (16)	26 (19)	
Gestational hypertension	93 (23)	32 (25)	33 (24)	28 (20)	
Preeclampsia	225 (56)	75 (58)	76 (56)	74 (53)	
Preeclampsia superimposed on chronic hypertension	22 (5)	5 (4)	6 (4)	11 (8)	
	Mean ± standard deviation				
**Maternal age at 6 months (years)**	34.9 ± 5.1	35.2 ± 5.3	34.5 ± 4.8	35.0 ± 5.4	0.56
	Number (%)				
**Ethnicity ^a^**					0.36
Caucasian	253 (63)	84 (65)	82 (60)	87 (63)	
Asian	73 (18)	23 (18)	29 (21)	21 (15)	
Aboriginal or Torres Strait Islander	2 (1)	0 (0)	1 (1)	1 (1)	
Polynesian	7 (2)	0 (0)	4 (3)	3 (2)	
European	28 (7)	12 (9)	8 (6)	8 (6)	
Middle Eastern	9 (2)	3 (2)	3 (2)	3 (2)	
African	8 (2)	4 (3)	1 (1)	3 (2)	
Other	12 (3)	2 (2)	6 (4)	4 (3)	
Mixed ethnicity	12 (3)	1 (1)	3 (2)	8 (6)	
**Maternal country of birth ^a^**					0.90
Australia	244 (60)	80 (62)	82 (60)	82 (60)	
Other	160 (40)	49 (38)	55 (40)	56 (40)	
**Highest level of formal education ^a^**					0.64
Secondary school	39 (10)	16 (12)	12 (9)	11 (8)	
Trade/Certificate/Diploma	82 (20)	23 (18)	27 (20)	32 (23)	
University degree	282 (70)	90 (70)	97 (71)	95 (69)	
**Partner ^a^**					0.98
Current partner not father of index baby	7 (2)	2 (2)	2 (2)	3 (2.2)	
Current partner is father of index baby	376 (93)	121 (94)	127 (93)	128 (93)	
Not applicable	21 (5)	6 (5)	8 (6)	7 (5)	
**Parity**					0.93
Index baby first baby	276 (68)	87 (67)	95 (69)	94 (68)	
Previous baby(ies) prior to index baby	129 (32)	42 (33)	42 (31)	45 (32)	
**Index pregnancy details**					0.91
Singleton	391 (97)	124 (96)	133 (97)	134 (96)	
Twins	14 (4)	5 (4)	4 (3)	5 (4)	
**Index pregnancy gestation at birth**					0.32
<37 weeks	114 (28)	41 (32)	40 (29)	33 (24)	
≥37 weeks	291 (72)	88 (68)	97 (71)	106 (76)	
**Maternal admission to acute care**					0.91
No	340 (84)	107 (83)	115 (84)	118 (85)	
Yes	65 (16)	22 (17)	22 (16)	21 (15)	
**Breastfeeding at 6 months postpartum ^a^**					0.15
Yes ^b^	263 (65)	89 (69)	93 (68)	81 (59)	
No	141 (35)	40 (31)	44 (32)	57 (41)	
	Median [IQR]				
**Maternal hospital length of stay (days)**	6.0 [5.0–8.0]	6.0 [5.0–8.0]	6.0 [4.5–8.0]	6.0 [5.0–8.0]	0.74

^a^ <1% missing data; ^b^ Includes breastfeeding exclusively, breastfeeding fully with occasional water and juices, and combination feeding. * *p* values determined using Pearson Chi-Square for categorical and ANOVA for continuous variables. Abbreviations: HDP, hypertensive disorders of pregnancy; *n*, number; IQR, interquartile range.

**Table 2:** Lifestyle outcomes at 6 and 12 months postpartum following hypertensive disorders of pregnancy.

	Overall (*n* = 405)			Group 1: Optimised Usual Care (*n* = 129)			Group 2: Brief Education Intervention (*n* = 137)			Group 3: Extended Lifestyle Intervention (*n* = 139)		
	6M	12M	*p*-Value	6M		6M	12M	*p*-Value	6M		6M	*p*-Value
Vegetables					Vegetables					Vegetables		
Serves/day, median [IQR]	2.0 [1.1–3.0]	2.5 [2.0–3.5]	**0.03**	2.5 [1.5–3.5]	Serves/day, median [IQR]	2.0 [1.1–3.0]	2.5 [2.0–3.5]	**0.03**	2.5 [1.5–3.5]	Serves/day, median [IQR]	2.0 [1.1–3.0]	**0.001**
Meeting recommended 5 serves/day, *n* (%)	39 (10)	35 (9)	0.66	14 (11)	Meeting recommended 5 serves/day, *n* (%)	39 (10)	35 (9)	0.66	14 (11)	Meeting recommended 5 serves/day, *n* (%)	39 (10)	0.50
Fruit					Fruit					Fruit		
Serves/day, median [IQR]	1.0 [1.0–2.0]	1.0 [1.0–2.0]	0.72	2.0 [1.0–2.0]	Serves/day, median [IQR]	1.0 [1.0–2.0]	1.0 [1.0–2.0]	0.72	2.0 [1.0–2.0]	Serves/day, median [IQR]	1.0 [1.0–2.0]	0.15
Meeting recommended 2 serves/day, *n* (%)	190 (47)	189 (47)	1.00	68 (53)	Meeting recommended 2 serves/day, *n* (%)	190 (47)	189 (47)	1.00	68 (53)	Meeting recommended 2 serves/day, *n* (%)	190 (47)	0.45
Moderate-vigorous activity *, mins/week, median [IQR]	280 [140–465]	280 [150–450]	0.78	275 [135–480]	Moderate-vigorous activity *, mins/week, median [IQR]	280 [140–465]	280 [150–450]	0.78	275 [135–480]	Moderate-vigorous activity *, mins/week, median [IQR]	280 [140–465]	0.21
Meeting recommended 150 min/week of moderate-vigorous exercise, *n* (%)	299 (74)	305 (76)	0.51	95 (74)	Meeting recommended 150 min/week of moderate-vigorous exercise, *n* (%)	299 (74)	305 (76)	0.51	95 (74)	Meeting recommended 150 min/week of moderate-vigorous exercise, *n* (%)	299 (74)	0.43
Physical activity, mins/week, median [IQR]					Physical activity, mins/week, median [IQR]					Physical activity, mins/week, median [IQR]		
Walking	180 [80–300]	150 [60–300]	0.26	190 [60–300]	Walking	180 [80–300]	150 [60–300]	0.26	190 [60–300]	Walking	180 [80–300]	0.94
Vigorous chores	0 [0–60]	15 [0–60]	0.20	10 [0–60]	Vigorous chores	0 [0–60]	15 [0–60]	0.20	10 [0–60]	Vigorous chores	0 [0–60]	0.09
Gardening/heavy yard work	0 [0–0]	0 [0–0]	0.82	0 [0–0]	Gardening/heavy yard work	0 [0–0]	0 [0–0]	0.82	0 [0–0]	Gardening/heavy yard work	0 [0–0]	0.67
Vigorous exercise	0 [0–60]	0 [0–60]	0.48	0 [0–60]	Vigorous exercise	0 [0–60]	0 [0–60]	0.48	0 [0–60]	Vigorous exercise	0 [0–60]	0.99
Moderate exercise	0 [0–0]	0 [0–0]	0.22	0 [0–0]	Moderate exercise	0 [0–0]	0 [0–0]	0.22	0 [0–0]	Moderate exercise	0 [0–0]	0.71
Strength exercise	0 [0–45]	0 [0–45]	0.16	0 [0–45]	Strength exercise	0 [0–45]	0 [0–45]	0.16	0 [0–45]	Strength exercise	0 [0–45]	0.06
Alcohol					Alcohol					Alcohol		
More than 4 standard drinks on at least one occasion in the past 4 weeks, *n* (%)	42 (10)	56 (14)	0.09	13 (10)	More than 4 standard drinks on at least one occasion in the past 4 weeks, *n* (%)	42 (10)	56 (14)	0.09	13 (10)	More than 4 standard drinks on at least one occasion in the past 4 weeks, *n* (%)	42 (10)	0.24
How often do you usually drink alcohol? *n* (%)			**0.01**		How often do you usually drink alcohol? *n* (%)	6 (2) 11 (3) 58 (14) 50 (12) 112 (28) 167 (41)		**0.01**	2 (2) 3 (2) 22 (17) 17 (13) 30 (23) 54 (42)	How often do you usually drink alcohol? *n* (%)		0.36
Daily	6 (2)	6 (2)		2 (2)	Daily		6 (2)			Daily	6 (2)	
4–6 times a week	11 (3)	14 (4)		3 (2)	4–6 times a week		14 (4)			4–6 times a week	11 (3)	
2–3 times a week	58 (14)	73 (18)		22 (17)	2–3 times a week		73 (18)			2–3 times a week	58 (14)	
Once a week	50 (12)	47 (12)		17 (13)	Once a week		47 (12)			Once a week	50 (12)	
Less than once a week	112 (28)	107 (27)		30 (23)	Less than once a week		107 (27)			Less than once a week	112 (28)	
Don’t drink alcohol	167 (41)	157 (39)		54 (42)	Don’t drink alcohol		157 (39)			Don’t drink alcohol	167 (41)	
Meat					Meat					Meat		
Processed meat serves/week, median [IQR]	1.0 [0.0–2.0]	1.0 [0.0–2.0]	**0.001**	1.0 [0.0–2.0]	Processed meat serves/week, median [IQR]	1.0 [0.0–2.0]	1.0 [0.0–2.0]	**0.001**	1.0 [0.0–2.0]	Processed meat serves/week, median [IQR]	1.0 [0.0–2.0]	0.14
Other food					Other food					Other food		
Hot fried potato serves/week, median [IQR]	1.0 [0.2–1.0]	0.5 [0.2–1.0]	**<0.001**	0.6 [0.0–1.0]	Hot fried potato serves/week, median [IQR]	1.0 [0.2–1.0]	0.5 [0.2–1.0]	**<0.001**	0.6 [0.0–1.0]	Hot fried potato serves/week, median [IQR]	1.0 [0.2–1.0]	**<0.001**
Salty snack serves/week, median [IQR]	0.5 [0.0–1.0]	0.2 [0.0–1.0]	**<0.001**	0.2 [0.0–1.0]	Salty snack serves/week, median [IQR]	0.5 [0.0–1.0]	0.2 [0.0–1.0]	**<0.001**	0.2 [0.0–1.0]	Salty snack serves/week, median [IQR]	0.5 [0.0–1.0]	**0.003**
Fast food occurrences/month, median [IQR]	2.0 [1.0–4.3]	2.0 [0.0–4.3]	**<0.001**	2.0 [1.0–4.3]	Fast food occurrences/month, median [IQR]	2.0 [1.0–4.3]	2.0 [0.0–4.3]	**<0.001**	2.0 [1.0–4.3]	Fast food occurrences/month, median [IQR]	2.0 [1.0–4.3]	**0.01**
What type of milk do you drink? *n* (%)			0.67		What type of milk do you drink? *n* (%)	198 (49) 207 (51)		0.67	61 (47) 68 (53)	What type of milk do you drink? *n* (%)		0.86
Full fat	198 (49)	193 (48)		61 (47)	Full fat		193 (48)			Full fat	198 (49)	
Other	207 (51)	212 (52)		68 (53)	Other		212 (52)			Other	207 (51)	
Juice and water, cups/week, median [IQR]					Juice and water, cups/week, median [IQR]					Juice and water, cups/week, median [IQR]		
Soft drink	0 [0.0–3.0]	0 [0.0–2.0]	**0.002**	0.0 [0.0–2.0]	Soft drink	0 [0.0–3.0]	0 [0.0–2.0]	**0.002**	0.0 [0.0–2.0]	Soft drink	0 [0.0–3.0]	**0.03**
Fruit juice	0 [0.0–2.0]	0 [0.0–1.0]	**<0.001**	0.0 [0.0–2.0]	Fruit juice	0 [0.0–2.0]	0 [0.0–1.0]	**<0.001**	0.0 [0.0–2.0]	Fruit juice	0 [0.0–2.0]	**0.02**
Water	7.0 [5.0–8.5]	6.0 [5.0–8.0]	**0.03**	7.0 [5.0–10.0]	Water	7.0 [5.0–8.5]	6.0 [5.0–8.0]	**0.03**	7.0 [5.0–10.0]	Water	7.0 [5.0–8.5]	0.53
How much does nutritional information influence the foods you purchase? *n* (%)			**<0.001**		How much does nutritional information influence the foods you purchase? *n* (%)	74 (18) 135 (33) 189 (47) 6 (2)		**<0.001**	18 (14) 33 (26) 75 (58) 3 (2)	How much does nutritional information influence the foods you purchase? *n* (%)		**<0.001**
Not at all	74 (18)	49 (12)		18 (14)	Not at all		49 (12)			Not at all	74 (18)	
A little	135 (33)	134 (33)		33 (26)	A little		134 (33)			A little	135 (33)	
A great deal	189 (47)	220 (54)		75 (58)	A great deal		220 (54)			A great deal	189 (47)	
Don’t know	6 (2)	2 (1)		3 (2)	Don’t know		2 (1)			Don’t know	6 (2)	

**Bold** = Significant at the *p* < 0.05 level. *p*-value calculated using McNemar’s test for categorical data and the Wilcoxon signed-rank test for non-parametric continuous data. * Calculated by the sum of walking, vigorous chores × 2, gardening/heavy yard work, vigorous exercise × 2, moderate exercise and strength exercise [20]. Abbreviations: 6M, 6 months; 12M, 12 months; *n*, number; IQR, interquartile range.

**Table 3:** Spearman’s rank correlations between change in lifestyle behaviours and change in cardiometabolic risk factors, stratified by BP.

Difference in Lifestyle Outcome	Normotensive (*n* = 317)			Hypertensive (SBP ≥ 140 or DBP ≥ 90) (*n* = 84)		
	SBP	DBP	BMI	SBP	DBP	BMI
Vegetables (serves/day)	0.06	−0.01	0.06	−0.35 **	−0.26 *	−0.10
Fruit (serves/day)	0.03	0.09	−0.01	−0.09	−0.10	−0.04
Total moderate-vigorous activity (mins/wk)	−0.06	−0.10	0.00	0.04	0.05	−0.05

**Bold** = Significant at the *p* < 0.05 level. ** Correlation is significant at the 0.01 level (2-tailed). * Correlation is significant at the 0.05 level (2-tailed). Abbreviations: SBP, systolic blood pressure; DBP, diastolic blood pressure; BMI, body mass index; mins, minutes; wk, week.

**Table 4:** Spearman’s rank correlations between change in lifestyle behaviours and change in cardiometabolic risk factors, stratified by BMI.

Difference in Lifestyle Outcome	Normal (BMI < 25 kg/m^2^) (*n* = 157)			Overweight (BMI 25–29.9 kg/m^2^) (*n* = 130)			Obese (BMI ≥30 kg/m^2^) (*n* = 117)		
	SBP	DBP	BMI	SBP	DBP	BMI	SBP	DBP	BMI
Vegetables (serves/day)	−0.04	−0.01	0.02	−0.02	−0.14	0.04	−0.02	−0.03	−0.02
Fruit (serves/day)	0.04	0.14	0.07	−0.09	−0.04	0.02	0.10	0.08	0.02
Total moderate-vigorous activity (mins/wk)	−0.05	−0.18 *	−0.08	0.02	0.04	0.04	−0.06	0.00	0.06

**Bold** = Significant at the *p* < 0.05 level. * Correlation is significant at the 0.05 level (2-tailed). Abbreviations: SBP, systolic blood pressure; DBP, diastolic blood pressure; BMI, body mass index; mins, minutes; wk, week.

## Data Availability

The data presented in this study are available on reasonable request from the corresponding author. The data are not publicly available due to ethics approval conditions.

## Supplementary Materials

The following supporting information can be downloaded at https://www.mdpi.com/article/10.3390/nu18040610/s1, Table S1: Lifestyle outcomes at 6 and 12 months postpartum following HDP, by HDP subtype; Table S2: Spearman’s rank correlations between change in lifestyle behaviours and change in cardiometabolic risk factors from 6 to 12 months postpartum following HDP.

## Abbreviations

The following abbreviations are used in this manuscript:

HDP	Hypertensive disorders of pregnancy
PE	Preeclampsia
GH	Gestational hypertension
CH	Chronic hypertension
PE+CH	Preeclampsia superimposed on chronic hypertension
CVD	Cardiovascular disease
BMI	Body mass index
BP	Blood pressure
SBP	Systolic blood pressure
DBP	Diastolic blood pressure
BP^2^	Blood Pressure Postpartum
ISSHP	International Society for the Study of Hypertension in Pregnancy
HH4M	Heart Health 4 Moms
RCT	Randomised controlled trial
6M	6 months
12M	12 months
