# Supplementary material for: Lifestyle Outcomes Six and Twelve Months After Hypertensive Disorders of Pregnancy: A Blood Pressure Postpartum Sub-Study
Source: Nutrients. 2026 Feb 12;18(4):610. doi: 10.3390/nu18040610 (PMC12943097; doi:10.3390/nu18040610)
Supplement: Supplementary file 1 [file nutrients-18-00610-s001.zip › nutrients-4026688-supplementary.pdf]

Supplementary Materials

Supplementary Table S1: Lifestyle outcomes at 6 and 12 months postpartum following HDP, by HDP subtype

	**Total (n=405)**			**CH (n=65)**			**GH (n=93)**			**PE (n=225)**			**PE+CH (n=22)**		
	6M	12M	p-value	6M	12M	p-value	6M	12M	p-value	6M	12M	p-value	6M	12M	p-value
**Alcohol**															
More than 4 standard drinks on at least one occasion in the past 4 weeks, n (%)	42 (10)	56 (14)	0.09	5 (8)	7 (11)	0.73	10 (11)	14 (15)	0.55	26 (12)	32 (14)	0.33	1 (5)	3 (14)	0.63
**How often do you usually drink alcohol? n (%)**			**0.01**			0.73			0.26			**0.02**			0.13
Daily	6 (2)	6 (2)		0	1 (2)		2 (2)	2 (2)		4 (2)	3 (1)		0	0	
4-6 times a week	11 (3)	14 (4)		2 (3)	1 (2)		4 (4)	4 (4)		4 (2)	8 (4)		1 (5)	1 (5)	
2-3 times a week	58 (14)	73 (18)		5 (8)	6 (9)		18 (19)	24 (26)		31 (14)	38 (17)		4 (18)	5 (23)	
Once a week	50 (12)	47 (12)		8 (12)	5 (8)		8 (9)	8 (9)		33 (15)	32 (14)		1 (5)	2 (9)	
Less than once a week	112 (28)	107 (27)		17 (26)	21 (32)		31 (33)	26 (28)		61 (27)	57 (25)		3 (14)	3 (14)	
Don't drink alcohol	167 (41)	157 (39)		33 (51)	31 (48)		30 (32)	29 (31)		91 (40)	86 (38)		13 (59)	11 (50)	
**Vegetables**															
Serves/day, median [IQR]	2.0 [1.1-3.0]	2.5 [2.0-3.5]	**0.03**	2.0 [1.0-3.0]	2.0 [1.0-3.0]	0.77	3.0 [1.8-4.0]	3.0 [2.0-4.0]	0.21	2.0 [1.3-3.0]	2.5 [2.0-3.5]	**0.05**	2.0 [1.0-4.0]	2.75 [2.0-4.0]	0.77
Meeting recommended 5 serves/day, n (%)	39 (10)	35 (9)	0.66	5 (8)	6 (10)	1.00	11 (12)	10 (11)	1.00	20 (9)	16 (7)	0.56	3 (14)	3 (14)	1.00
**Fruit**															
Serves/day, median [IQR]	1.0 [1.0-2.0]	1.0 [1.0-2.0]	0.72	1.0 [0.4-2.0]	1.0 [1.0-2.0]	0.06	2.0 [1.0-2.0]	1.0 [1.0-2.0]	0.61	2.0 [1.0-2.0]	1.5 [1.0-2.0]	0.74	1.0 [0.8-2.0]	1.0 [1.0-2.0]	0.29
Meeting recommended 2 serves/day, n (%)	190 (47)	189 (47)	1.00	21 (32)	27 (42)	0.24	47 (51)	43 (46)	0.63	116 (52)	110 (49)	0.62	6 (27)	9 (41)	0.45
**Meat**															
Processed meat serves/week, median [IQR]	1.0 [0.0-2.0]	1.0 [0.0-2.0]	**0.001**	1.0 [0.0-3.0]	1.0 [0.0-2.5]	0.70	1.0 [0.0-2.0]	1.0 [0.0-2.0]	0.31	1.0 [0.0-2.0]	1.0 [0.0-2.0]	**0.001**	1.0 [0.0-2.9]	1.0 [0.0-2.0]	0.28
**Other food**															
Hot fried potato serves/week, median [IQR]	1.0 [0.2-1.0]	0.5 [0.2-1.0]	**<0.001**	1.0 [0.2-2.0]	1.0 [0.0-1.0]	**0.03**	1.0 [0.2-1.0]	0.5 [0.1-1.0]	0.07	1.0 [0.2-1.0]	0.5 [0.2-1.0]	**<0.001**	0.6 [0.0-2.0]	1.0 [0.3-1.1]	0.53
Salty snack serves/week, median [IQR]	0.5 [0.0-1.0]	0.2 [0.0-1.0]	**<0.001**	0.5 [0.0-1.0]	0.2 [0.0-1.0]	0.26	0.5 [0.0-1.3]	0.2 [0.0-1.0]	**0.01**	0.5 [0.0-1.0]	0.2 [0.0-1.0]	**0.01**	1.0 [0.0-1.0]	0.2 [0.0-2.0]	0.39
Fast food occurrences/month, median [IQR]	2.0 [1.0-4.3]	2.0 [0.0-4.3]	**<0.001**	4.3 [2.0-4.3]	2.0 [1.0-4.3]	**0.01**	2.0 [1.0-4.3]	2.0 [0.0-4.3]	**0.03**	2.0 [1.0-4.3]	2.0 [0.0-4.3]	**0.02**	1.8 [0.0-5.4]	2.0 [1.0-4.3]	0.76
**What type of milk do you drink? n (%)**			0.67			0.27			0.61			1.00			0.63
Full fat	198 (49)	193 (48)		43 (66)	38 (59)		39 (42)	42 (45)		103 (46)	102 (45)		13 (59)	11 (50)	
Other	207 (51)	212 (52)		22 (34)	27 (42)		54 (58)	51 (55)		122 (54)	123 (55)		9 (41)	11 (50)	
**Juice and water (cups/week, median [IQR])**	0 [0.0-3.0]	0 [0.0-2.0]	**0.002**	2.0 [0.0-6.8]	1.0 [0.0-5.0]	0.13	0 [0.0-2.0]	0 [0.0-1.8]	0.12	0 [0.0-2.0]	0 [0.0-2.0]	0.09	0.3 [0.0-3.3]	0 [0.0-4.0]	0.07
Soft drink	0 [0.0-2.0]	0 [0.0-1.0]	**<0.001**	0 [0.0-3.0]	0 [0.0-1.5]	0.14	0 [0.0-1.5]	0 [0.0-0.9]	**0.004**	0 [0.0-2.0]	0 [0.0-1.0]	**<0.001**	0 [0.0-1.0]	0 [0.0-0.4]	0.28
Fruit juice	7.0 [5.0-8.5]	6.0 [5.0-8.0]	**0.03**	7.0 [4.0-8.0]	7.0 [4.5-8.0]	0.83	7.0 [5.0-9.0]	6.0 [5.0-8.0]	0.07	7.0 [5.0-8.5]	6.0 [5.0-8.0]	0.13	8.0 [5.8-10.0]	7.5 [4.9-9.3]	0.78

Water															
**How much does nutritional information influence the foods you purchase? n (%)**			**<0.001**			**0.01**			0.12			**0.01**			0.41
Not at all	74 (18)	49 (12)		21 (33)	11 (17)		14 (15)	8 (9)		38 (17)	27 (12)		1 (5)	3 (14)	
A little	135 (33)	134 (33)		20 (31)	25 (39)		25 (27)	27 (29)		80 (36)	76 (34)		10 (46)	6 (27)	
A great deal	189 (47)	220 (54)		21 (33)	29 (45)		53 (57)	58 (62)		105 (47)	120 (53)		10 (46)	13 (59)	
Don't know	6 (2)	2 (1)		2 (3)	0 (0)		1 (1)	0 (0)		2 (1)	2 (1)		1 (5)	0 (0)	
**Physical activity (mins/week, median [IQR])**															
Walking	180 [80-300]	150 [60-300]	0.26	120 [43-290]	120 [30-225]	0.71	190 [90-300]	150 [68-240]	**0.04**	180 [70-300]	180 [88-320]	0.53	190 [90-375]	140 [60-240]	0.07
Vigorous chores	0 [0-60]	15 [0-60]	0.20	5 [0-101]	0 [0-60]	0.22	0 [0-53]	0 [0-60]	0.61	0 [0-60]	15 [0-60]	0.13	18 [0-75]	40 [0-120]	0.09
Gardening/heavy yard work	0 [0-0]	0 [0-0]	0.82	0 [0-0]	0 [0-0]	0.81	0 [0-0]	0 [0-0]	0.92	0 [0-0]	0 [0-0]	0.89	0 [0-0]	0 [0-0]	0.60
Vigorous exercise	0 [0-60]	0 [0-60]	0.48	0 [0-30]	0 [0-60]	0.29	0 [0-103]	0 [0-58]	0.06	0 [0-60]	0 [0-60]	0.20	0 [0-2.5]	0 [0-135]	0.26
Moderate exercise	0 [0-0]	0 [0-0]	0.22	0 [0-0]	0 [0-0]	0.44	0 [0-0]	0 [0-0]	0.86	0 [0-0]	0 [0-0]	0.28	0 [0-0]	0 [0-0]	0.41
Strength exercise	0 [0-45]	0 [0-45]	0.16	0 [0-46]	0 [0-60]	0.27	0 [0-60]	0 [0-53]	**0.03**	0 [0-45]	0 [0-40]	0.29	0 [0-5]	0 [0-8]	0.67
**Moderate-vigorous activity* (mins/week, median [IQR])**	280 [140-465]	280 [150-450]	0.78	245 [120-476]	280 [93-405]	0.74	300 [150-480]	270 [143-420]	0.06	270 [140-450]	285 [150-490]	0.09	340 [130-473]	290 [188-493]	0.51
**Meeting recommended 150 min/week of moderate-vigorous exercise, n (%)**	299 (74)	305 (76)	0.51	45 (69)	45 (69)	1.00	72 (77)	69 (74)	0.86	167 (74)	172 (76)	0.50	15 (68)	19 (86)	0.22

Bold = Significant at the $p<0.05$ level.
* Calculated by the sum of walking, vigorous chores*2, gardening/heavy yard work, vigorous exercise*2, moderate exercise and strength exercise[20].
Abbreviations: HDP, hypertensive disorders of pregnancy; 6M, 6 months; 12M, 12 months; CH, chronic hypertension; GH, gestational hypertension; PE, preeclampsia; PE+CH, preeclampsia superimposed on chronic hypertension; n, number; IQR, interquartile range.

Supplementary Table S2: Spearman's rank correlations between change in lifestyle behaviours and change in cardiometabolic risk factors from 6 to 12 months postpartum following HDP.

**Difference in lifestyle outcome**	**Difference in SBP**	**Difference in DBP**	**Difference in BMI**
Vegetables (serves/day)	-0.03	-0.06	0.01
Fruit (serves/day)	0.02	0.06	-0.01
Processed meat (serves/wk)	-0.09	-0.05	-0.07
Hot fried potato product (serves/wk)	0.06	0.01	-0.06
Salty snack (serves/wk)	-0.05	-0.06	-0.06
Soft drink (cups/wk)	-0.01	-0.02	-0.06
Fruit juice (cups/wk)	0.07	0.08	-0.001
Water (cups/day)	-0.01	0.03	-0.03
Walking (mins/wk)	-0.01	-0.08	0.05
Vigorous chores (mins/wk)	-0.04	0.01	-0.02
Gardening/heavy yard work (mins/wk)	0.02	-0.01	-0.07
Vigorous exercise (mins/wk)	-0.04	0.05	0.001
Moderate exercise (mins/wk)	-0.02	0.02	-0.08
Strength exercise (mins/wk)	-0.05	-0.07	-0.03
Total moderate-vigorous activity (mins/wk)	-0.03	-0.05	-0.002

Abbreviations: HDP, hypertensive disorders of pregnancy; SBP, systolic blood pressure; DBP, diastolic blood pressure; BMI, body mass index; wk, week.
